# *TP53inp1* Gene Is Implicated in Early Radiation Response in Human Fibroblast Cells

**DOI:** 10.3390/ijms161025450

**Published:** 2015-10-23

**Authors:** Nikolett Sándor, Boglárka Schilling-Tóth, Enikő Kis, Lili Fodor, Fruzsina Mucsányi, Géza Sáfrány, Hargita Hegyesi

**Affiliations:** 1 Division of Molecular Radiobiology, National Public Health Center—National Research Directorate for Radiobiology and Radiohygiene, Anna 5, Budapest 1221, Hungary; E-Mails: sandor.nikolett@osski.hu (N.S.); schilling.toth.boglarka@osski.hu (B.S.-T.); kis.eniko@osski.hu (E.K.); safrany.geza@osski.hu (G.S.); 2 Doctoral School of Pathological Sciences, Semmelweis University, Üllői 26, Budapest 1089, Hungary; 3 Department of Genetics, Cell and Immunobiology, Semmelweis University, Nagyvárad tér 4, Budapest 1089, Hungary; E-Mails: fodorlilierika@gmail.com (L.F.); fruzsimu@gmail.com (F.M.); 4 Department of Morphology and Physiology, College of Health Care, Semmelweis University, Vas 17, Budapest 1089, Hungary

**Keywords:** *TP53inp1*, p53-network, autophagy, senescence, radiosensitivity, RNA interference, *GDF-15*, *CDKN1A*

## Abstract

Tumor protein 53-induced nuclear protein-1 (*TP53inp1*) is expressed by activation via p53 and p73. The purpose of our study was to investigate the role of *TP53inp1* in response of fibroblasts to ionizing radiation. γ-Ray radiation dose-dependently induces the expression of *TP53inp1* in human immortalized fibroblast (F11hT) cells. Stable silencing of *TP53inp1* was done via lentiviral transfection of shRNA in F11hT cells. After irradiation the clonogenic survival of *TP53inp1* knockdown (F11hT-shTP) cells was compared to cells transfected with non-targeting (NT) shRNA. Radiation-induced senescence was measured by SA-β-Gal staining and autophagy was detected by Acridine Orange dye and microtubule-associated protein-1 light chain 3 (LC3B) immunostaining. The expression of *TP53inp1*, *GDF-15*, and *CDKN1A* and alterations in radiation induced mitochondrial DNA deletions were evaluated by qPCR. *TP53inp1* was required for radiation (IR) induced maximal elevation of *CDKN1A* and *GDF-15* expressions. Mitochondrial DNA deletions were increased and autophagy was deregulated following irradiation in the absence of *TP53inp1*. Finally, we showed that silencing of *TP53inp1* enhances the radiation sensitivity of fibroblast cells. These data suggest functional roles for *TP53inp1* in radiation-induced autophagy and survival. Taken together, we suppose that silencing of *TP53inp1* leads radiation induced autophagy impairment and induces accumulation of damaged mitochondria in primary human fibroblasts.

## 1. Introduction

Ionizing radiation (IR) causes oxidative stress in DNA, proteins and lipids, but cells have a complex signal cascade to avoid ROS-induced damage and ensure their homeostasis. Dependent on the extent of radiation damage and the genetic background of cells, signal molecules trigger cell cycle arrest and DNA repair, or in the case of lethal/sub-lethal damage, elimination of the cells by senescence or apoptosis. Oxidative damage response is regulated by p53 and its cofactors; they have a critical role in the outcome of cell injury [1]. *TP53inp1* is one of the downstream target of p53/p73 and it also has a feedback regulation to p53 and it stimulates their capacity to control cell cycle [2,3]. *TP53inp1*α and *TP53inp1*β isoforms are encoded by the *TP53inp1* gene [4]. It is known that *TP53inp1* acts as an antioxidant and promotes caspase-dependent apoptosis [5]. It was recently shown that TP53inp1-dependent apoptosis was mediated by homeodomain-interacting protein kinase-2 (HIPK2), via p53 [6]. One of the key consequences of exposures of different cells to ionizing radiation is the change in the expression level of multiple genes [7,8]. In normal human (fibroblast) cells several ataxia telangiectasia mutated (ATM)/p53 associated genes such as *TP53inp1*, *CDKN1A*, and *HDM2*, as well as several tumor necrosis factor (TNF) receptor superfamily members were shown to be induced by IR [9,10]. Many authors report that *TP53inp1* has a role in the control of proliferation and apoptosis under stress condition and acts as a dual regulator of transcription and autophagy [11], but the precise role of *TP53inp1* in the radiation induced cellular stress remains ambiguous. In the recent work, we show evidence of the dose-dependent transcription of *TP53inp1* by IR. Until now, it is not yet known whether the level of *TP53inp1* expression can affect the radiosensitivity of human fibroblasts and whether TP53inp1 can modify the effect of radiotherapy. Thus, we established a shRNA-mediated *TP53inp1* silencing strategy to investigate the effect of *TP53inp1* silencing on cell survival and sensitization to γ-radiation in human fibroblasts *in vitro*.

## 2. Results

### 2.1. TP53inp1 Is a Radiation Response Gene in Fibroblast Cells

**Figure 1:**
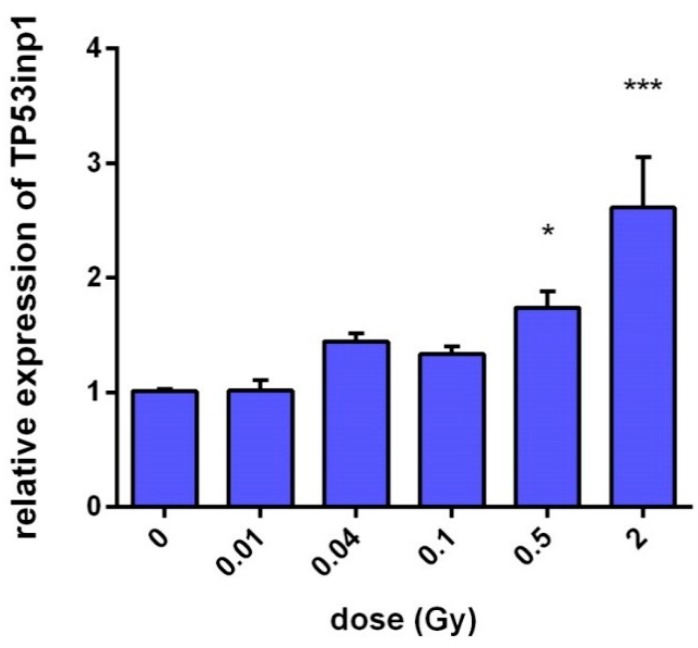
Dose-dependent expression of *TP53inp1* in immortalized human fibroblast cells (F11hT). Relative gene expression was measured by qPCR with the delta-delta cycle threshold (ΔΔ*C*_t_) method as described in the Experimental Section. The data are derived from at least three independent experiments, and error bars show SEM of the mean. Gene expression in the F11hT cells is expressed in comparison with the sham-irradiated fibroblasts cells (calibrators), in which levels of expression are regarded as a level of one. Cells were harvested 2 h after γ-irradiation. One-way ANOVA was used for analysis. (* *p* < 0.05, *** *p* < 0.001).

**Figure 2:**
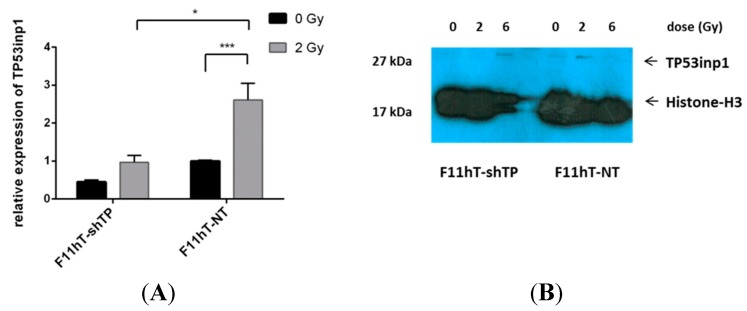
*TP53inp1* gene silencing in F11hT-NT and F11hT-shTP cells. (**A**) Values were calculated by qPCR with the ΔΔCT method. Data are given from at least four experiments, and error bars show SEM of the mean. Gene expression in the F11hT-shTP cells is compared with the sham-irradiated F11ht-NT cells, where the expression is fixed as a level of one. Statistical analysis was performed using one-way ANOVA-test (* *p* < 0.05, *** *p* < 0.001). (**B**) Irradiation induces expression of *TP53inp1*. *TP53inp1* protein level was detected by Western blot at 24h post-irradiation with 2 and 6 Gy and normalized to Histone-H3. Expression of *TP53inp1* protein was significantly lower in *TP53inp1* silenced F11hT-shTP cells as compared to the F11hT-NT cells. Densitometric analysis of the bands, relative to Histone-H3, was performed using ImageJ softwer (http://imagej.nih.gov/ij/).

The dose-dependent radiation-induced gene expression of the *TP53inp1* gene was measured in irradiated F11hT human fibroblast cells by quantitative polymerase chain reaction (qPCR). In irradiated cells expression of *TP53inp1* increased with dose 2 h after irradiation (Figure 1). Elevation of *TP53inp1* was obtained from 100 mGy (1.33 ± 0.12, *p* = 0.059), although the alterations became statistically significant only above 500 mGy (1.74 ± 0.25, *p* = 0.027). Treatment with 2 Gy further increased the expression of *TP53inp1* up to (2.613 ± 0.439, *p* = 0.025). The expression of *TP53inp1* protein was also elevated 24 h post-irradiation (Figure 2B) in human immortalized fibroblast (F11hT-NT).

### 2.2. Lentiviral Delivery of TP53inp1-Targeting shRNA Effectively Decreases TP53inp1 Expression and Increases Radiation Sensitivity

It was shown that high-efficiency RNA interference can be accomplished by overexpressing an exogenous shRNA that has been engineered to encode a 19–25 base pair sequence that complements a segment of the gene targeted for knockdown [12]. In the present study we have attempted to silence the *TP53inp1* gene by lentiviral shRNAs as described in the Experimental Section. The efficiency of *TP53inp1* mRNA level knockdown was verified by qPCR in F11hT-NT and F11hT-shTP cells both in their normal growth state and after 2 Gy irradiations (Figure 2A). Silencing TP53inp1 with shRNA effectively decreased *TP53inp1* mRNA expression by 65%–90% (*p* < 0.01) in F11hT-shTP cells. Expression levels of *TP53inp1* increased slightly in the F11ht-NT cells at 2 h after 2 Gy irradiation. As shown in Figure 2B, an increase in *TP53inp1* was also detected on protein level in the 2 Gy exposed F11hT-NT group compared with the non-irradiated controls. By contrast, there were almost no detectable *TP53inp1* proteins in the *TP53inp1* silenced F11hT-shTP non-irradiated group; moreover, the 2 Gy-induced elevation was less than in F11hT-NT cells (Figure 2B). Density of bands was normalized to Histone-H3 by densitometry analysis; the data are given in pixel density of TP53inp1/Histone-H3 (F11hT-shTP 0 Gy: 0.006; 2 Gy: 0.001; 6 Gy: 0.042; F11hT-NT 0 Gy: 0.020; 2 Gy: 0.064; 6 Gy: 0.021).

**Figure 3:**
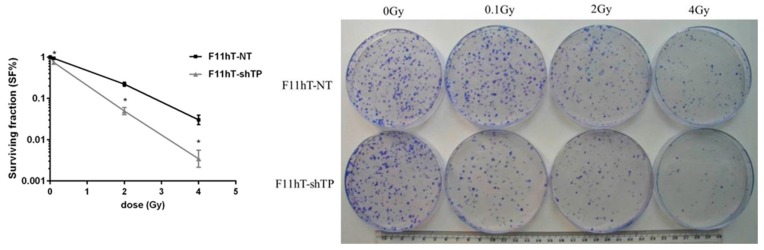
Radiation survival curve of the F11hT-NT and F11hT-shTP cell lines. On the **left** panel quantitative analysis is shown. Data are based on at least six independent experiments, and error bars represent SEM of the mean survival following exposure to 0, 0.1, 2, and 4 Gy γ-radiation. SF% was calculated according to the following formula: SF% = (PE of treated sample/PE control sample) X 100. PE: plating efficiency. Welch’s test was used for statistical evaluation (* *p* < 0.05); The **right** panel shows representative Coomassie BB stained colonies. For the colony-forming assay, 1500 cells were seeded on 10 cm diameter Petri dishes and irradiated with 0.1-, 2-, and 4 Gy γ-rays. The upper series are representing the F11hT-NT fibroblasts, while the lower panel shows the F11hT-shTP cells.

Next, we looked whether silencing of *TP53inp1* could affect radiation-induced cell death. F11hT-NT and F11hT-shTP cells were irradiated and grown for 14 days and the survival colonies was counted. F11hT-shTP cells formed fewer colonies after irradiation than F11hT-NT cells transfected with the non-targeted (NT) vector (Figure 3). Silencing *TP53inp1* causes increased radiosensitivity.

### 2.3. TP53inp1 Is Implicated in Autophagy

**Figure 4:**
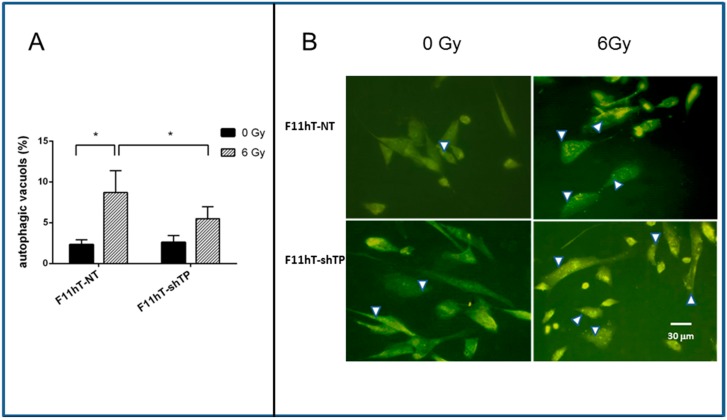

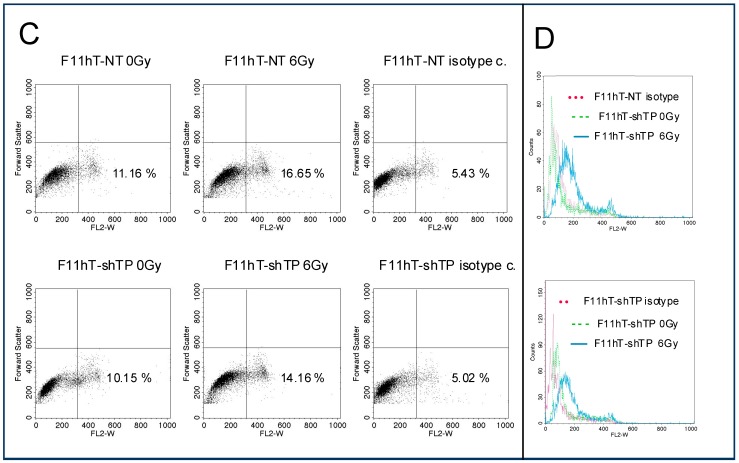
The effect of *TP53inp1* silencing on the formation of radiation-induced autophagic vacuoles. (**A**) Quantitation of autophagic vacuoles shows a significant increase in the 6 Gy treated groups as compared to the sham irradiated F11hT-NT cells (* *p* value < 0.5). Silencing of *TP53inp1* is resulted significantly less autophagosome in 6 Gy-exposed F11hT-shTP cells (* *p* value < 0.5). Two days after irradiation, cells were treated with Acridine Orange dye and red (autophagosome) puncta were counted from minimum eight cover slips (*n* ≥ 8) under fluorescent microscope. White arrowheads denote the autophagic vacuoles. Results were analyzed with One-way ANOVA; (**B**) fluorescence photomicrograps obtained after Acridine Orange staining. Control cells (0 Gy) showing a few cytoplasmic AV formation, the number of AV increased in irradiated F11hT-NT cells and, to a lesser extent, in F11hT-shTP cells; (**C**) Representative flow cytometry plots are demonstrative of LC3B intracellular staining in response to 6 Gy exposures. Dot plot analysis is derived from the non-gated cell population. Flow cytometry analysis of F11hT-NT and F11hT-shTP cells using LC3B Antibody (Sigma, St. Louis, MI, USA) compared to a nonspecific isotype control antibody. Acquisition of 10,000 events was collected and for analysis the CellQuest software (BD Biosciences, San Jose, CA, USA) was used; (**D**) Representative flow cytometry histograms of percent LC3B-positive fibroblast are shown at right. Labeling of LC3B-positive cells at 48 h in F11hT-NT cells (right, top graph) and F11hT-shTP cells (right, bottom graph) after irradiation are graphed.

We analyzed whether the increased radiation sensitivity of F11hT-shTP cells is related to higher rates of autophagy. F11hT-NT and F11hT-shTP cells were irradiated by 6 Gy and autophagy vacuole formations were monitored by Acridine Orange (AO) staining followed by fluorescent microscopy. The quantified number of AO-positive vacuoles is shown in Figure 4A. The percentage of AO-positive vacuoles is increased in F11hT-NT (2.333 ± 0.589) cells treated with 6 Gy (8.718 ± 2.66) compared to untreated cells demonstrating that IR induces the accumulation of autophagic vacuoles (AV) in F11hT-NT cells. Stable silencing of *TP53inp1* markedly reduced the number of radiation-induced autophage vacuoli in F11hT-shTP cells compared to F11hT-NT (5.506 ± 1.469, 8.718 ± 2.66, respectively) (Figure 4A,B).

To assess the development of AVs in *TP53inp1* silenced fibroblasts, we also performed immunostaining with LC3B antibody and quantified the result with flow cytometry (Figure 4C,D) [13]. The percentage of LC3B-positive AV dots was significantly increased from 11.6% to 16.65% by 6 Gy exposure in F11hT-NT cells, whereas there was less increase in the percentage of F11hT-shTP with LC3B-positive AV dots (from 10.15% to 14.16%) (Figure 4C). The values of fluorescence intensity are taken from the modes of the FACS histograms shown in the right panel (Figure 4D). These results suggest that *TP53inp1* might contribute to the formation of radiation-induced autophagy in human fibroblasts.

### 2.4. TP53inp1 Enhances the Accumulation of Common Deletion (CD) in Mitochondrial Genome

**Figure 5:**
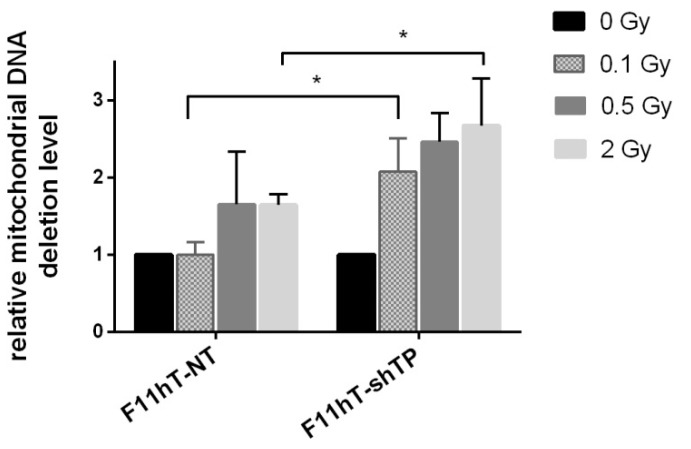
Effect of *TP53inp1* silencing on the accumulation of CD (common deletion) in the mitochondrial genome. Dose-dependent increase of mitochondrial common DNA deletions was compared in irradiated F11hT-NT and F11hT-shTP cells by qPCR. The mean ± SEM of at least three independent experiments are shown. Changes in the relative amount of CD were measured 72 h after the γ-irradiation. The mean ± SEM data derived from at least three experiments. Statistically significant changes calculated with One-way ANOVA are labeled as * *p* < 0.05.

The effect of *TP53inp1* silencing on the radiation response of fibroblast cells was investigated by the analysis of common deletions in the mitochondrial genome. F11hT-NT cells showed accumulation of CD, dose-dependently, from 2 Gy (1.647 ± 0.413; *p* < 0.05) doses. In F11hT-shTP cells the IR-induced effect was more pronounced at 0.1 Gy, (2.080 ± 0.420; *p* < 0.05) and also at 2 Gy (2.673 ± 0.61; *p* < 0.051) at 48 h post-irradiation (Figure 5). The data might suggest that the elimination of mutated mitochondria were impaired in *TP53inp1* silenced cells.

**Figure 6:**
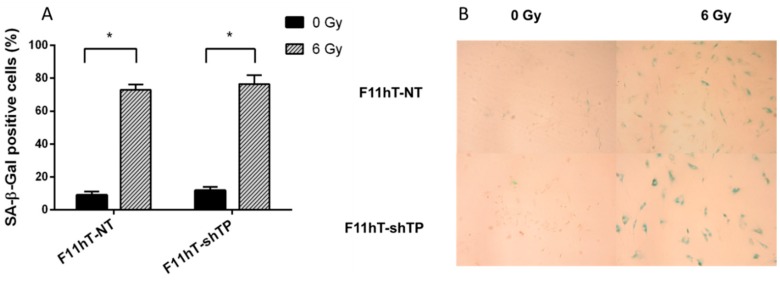
(**A**) Effect of *TP53inp1* silencing on radiation induced senescence. Senescence associated-β-galactosidase positive F11hT-NT and F11hT-shTP cells was measured six days after exposure to a single dose of 6 Gy irradiation. Data presented are means ± SEM, *n* = 9 from three separate experiments. Statistical analysis was performed with one-way ANOVA followed by a Bonferroni post-test. A statistically significant difference *p* < 0.05 (*) is indicated; (**B**) Representative pictures of human fibroblasts (F11hT-NT) and *TP53inp1* silenced fibroblasts (F11hT-shTP) were irradiated with 6Gy and stained with SA-βGal. The sham irradiated control shows exiguous staining, while the 6 Gy irradiated samples are powerfully stained. (Photo: Zeiss Axioskop2plus microscope, 100× magnification; Olympus Camedia camera; 3× optical zoom).

### 2.5. TP53inp1 Does Not Regulate IR-Induced Cellular Senescence

Next, we investigated whether down-regulated *TP53inp1* might play a role in regulation of IR-induced premature senescence. Thus, we performed a loss-of-function analysis of *TP53inp1* by stable transfecting F11hT cells with *TP53inp1*-shRNA to evaluate their effects on IR-induced senescence. Our data indicate that SA-β-gal stained cells are increased in F11hT-NT cells (8.944 ± 2.2757; 72.972 ± 3.182; *p* < 0.05) and F11hT-shTP cells (11.791 ± 2.211, 76.468 ± 5.425 *p* < 0.05) in 6 Gy treated cultures at 6 days post-irradiation, but there were no difference between treated F11hT-NT and F11hT-shTP cells, suggesting that silencing of *TP53inp1* does not modifies IR-induced senescence in human fibroblast cells (Figure 6A,B).

### 2.6. TP53inp1 Modify the Radiation-Induced Expression of CDKN1A and GDF-15 Gene

**Figure 7:**
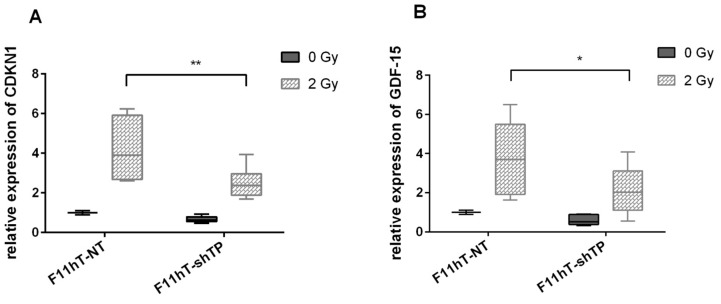
*TP53inp1* silencing alters expression of IR–induced p53 targets. Graphs show relative transcript expression of *CDKN1A* in (**A**) panel GDF-15 and in (**B**) panel, as quantified by qPCR in F11hT-NT and F11hT-shTP fibroblasts without treatment and after 2 Gy γ ray exposure for 2 h (* and ** are *p* < 0.05 and 0.01 compared with treated F11hT-NT and F11hT-shTP cells, respectively). Target transcript expression was normalized by the corresponding mean of housekeeping GAPDH and β-Actin values. Data are means of triplicates ± SEM. Statistically significant changes calculated with One-way ANOVA are labelled as * *p* < 0.05.

In a previous publication, we showed that both *CDKN1A* and *GDF-15* mRNAs were induced *in vitro* in human fibroblast cells by low doses of γ-rays [8]. Exposure from 0.1 Gy resulted in a significant induction for both genes. Therefore, the expression levels of *CDKN1A* and *GDF-15* mRNAs were compared after irradiation with 2 Gy in F11hT-NT and F11hT-shTP cells. RNAs were isolated from control and irradiated cells 2 h after irradiation, in order to quantify the expressions of *CDKN1A*, and *GDF-15* by qPCR. Significant induction of both radiation response genes were observed after irradiation with 2 Gy (1.0 ± 0.01, 4.166 ± 0.867 and 1.0 ± 0.01, 3.788 ± 0.758, *p* < 0.01) (Figure 7A,B). The expression of *CDKN1A* and GDF-15 mRNAs reduced significantly in *TP53inp1* silenced F11hT-shTP cells exposed to 2 Gy (0.672 ± 0.05, 2.516 ± 0.226 and 0.611 ± 0.119, 2.084 ± 0.332, *p* < 0.05) (Figure 5A,B).

## 3. Discussion

Previously, we had identified several early radiation response genes in irradiated primary human fibroblast cells, among them the currently investigated *TP53inp1* [10]. Here we further explored the potential role of *TP53inp1* in the response of fibroblast cells to ionizing radiation. *TP53inp1* is an oxidative damage-induced protein that is a downstream target of p53 [2,14]. It was previously mentioned that it is a major mediator of p53 antioxidant function [15]. Very interestingly *TP53inp1* is not only controlled by p53 but, on the other hand, it might alter p53 transcriptional activity on several p53-dependent promoters, thus stimulating the capacity of p53 to induce cell death [6,16]. *TP53inp1* might also act independently from p53; in the absence of p53 *TP53inp1* with p73 regulates cell cycle delay and apoptosis [3].

The current data indicate that *TP53inp1* has a crucial role in redox homeostasis it inhibits proliferation, and enhances apoptosis and the expression of it is reduced in many solid tumors [17,18,19]. Our results have indicated that treatment of F11hT cells with γ-radiation dose-dependently induces the expression of *TP53inp1*. These results suggest that increased expression of *T53inp1* is involved in the radiation-induced stress response in normal human fibroblasts. Previous observation suggested that *TP53inp1* expression increases in inflammation or other stress agents, such as ionizing and non-ionizing radiation [10,20,21].

In order to evaluate the role of *TP53inp1* in radiation response we have silenced the *TP53inp1* gene by the stable introduction of shRNAs into immortalized human skin fibroblast cells. Our data indicate that silencing of *TP53inp1* enhances the radiation sensitivity of fibroblast cells. That means that a higher percentage of silenced cells survive a given radiation dose. It is well-known that radiation-induced cell death is a complex process. Cells might die by apoptosis, necrosis autophagy, and senescence, and by mitotic catastrophe. Usually, not the type, but the timing of the cell death is the most important process. Cells might die early after irradiation or they can undergo late cell death [22]. Most of the normal human cells suffer late cell death; they die days, or even weeks, after irradiation. Previously, it was reported that knockdown of *TP53inp1* suppressed the growth of cervical cancer cells and promoted apoptosis [23] in Hela cells. However, primary human fibroblast cells are not capable for radiation-induced apoptosis; they mostly die by mitotic catastrophe and senescence. The contribution of these pathways to radiation-induced cell death depends on individual factors [24]. Our current data indicate that *TP53inp1* silencing has no effect on radiation-induced senescence in telomerase immortalized human fibroblast cells. In this extent, it was recently shown that human embryonic lung diploid fibroblasts cells (WI-38) transfected with *TP53inp1* siRNA had significantly reduced IR-induced cellular senescence [24]. We assume that individual differences in the applied cell lines might explain the different response.

Continuously accumulating data suggest that the autophagic response of normal and cancer cells to IR is one of the major pathways that leads to cell death [25]. Previously, it was shown that radiation-induced autophagy and senescence may occur independently, because senescence can develop when autophagy is impaired [26]. There are indications in the scientific literature that *TP53inp1* is associated with autophagy; it interacts with the ATG8 family of proteins and promotes autophagy-dependent cell death [27]. Therefore, we decided to study radiation-induced autophagic responses in *TP53inp1*-silenced cells. We performed autophagy assays on F11hT cells and cell with stably expressing *TP53inp1* shRNA. In the standard cultivation, the number of autophagosomes was low, and similar in F11hT-NT and F11hT-shTP cells. As expected, radiation treatment triggered autophagy in both cell lines as demonstrated previously in glioblastoma cell lines by others [28]. However, the number of AV-positive cells was almost cut in half in silenced cells, suggesting that *TP53inp1* silencing inhibits the formation of autophagy, it is in line with the results of Seillier M. *et al.* who demonstrated that *TP53inp1* was crucial for the autophagic activity of exposed cells [27]. However, the decreased authopagy in *TP53inp1* silenced cells does not explain, but contradicts, the increased radiation sensitivity of silenced cells. Chang *et al.* [29] suggested recently that the reduction in autophagy might correlate with increased apoptosis induction and by suppression of the NHEJ and HR repair pathways. There are also some evidence that in normal cells autophagy might be cytoprotective [30,31]. We might hypothesize that *TP53inp1*-mediated autophagy could be part of the cellular response against oxidative stress that protects against cell organelle failure by inducing death of cells with excessive radiation damage. Impaired autophagy can induce ROS accumulation and DNA damage [32]. *TP53inp1* could be a partner in displacing p62 from LC3, therefore enhancing degradation of proteins, for example, antiapoptotic proteins, whose depletion would lead to cell death [4]. At present, the role of autophagy in cell death and radiosensitivity remains controversial [33,34]; probably, in the presence of decreased autophagy other cellular death pathways, such as mitotic catastrophe, are activated.

We have also investigated the effect of *TP53inp1* silencing on the development of radiation-induced mitochondrial DNA damage. The data demonstrated that *TP53inp1* silencing increased the number of CD in mitochondria 48 h after irradiation. Basically, it is in concordance with increased radiation sensitivity—decreased survival—of silenced cells suggesting that radiation-induced damage is less efficiently repaired both in the genomic and mitochondrial DNAs. However, one might also find correlations among increased CD frequency and decreased autophagy in *TP53inp1-*silenced cells. Decreased autophagy might mean the less efficient removal of damaged mitochondria from the cells. In line with this hypothesis there are several recent reports suggesting that induction of autophagy, and the degradation and elimination of mutated mitochondria are correlated [35,36,37].

Investigating potential correlations between gene expression and specific radiation injury or long-term outcomes like carcinogenesis have an upmost importance. The p53 tumor suppressor protein has the basic role in regulating cellular radiation response [38]. As mentioned before expression of *TP53inp1* is controlled by p53 and on the other hand *TP53inp1* might also have influences on the effect of p53 on the other p53 downstream targets. Our experiments demonstrated that *TP53inp1* silencing modulates the transcription of such known p53 pathway genes as CDKN1A and GDF-15 Formerly, these genes were also identified among the consensus radiation response genes by us [10]. Others also reported that *TP53inp1* transcriptional induction upon stress was associated with an increased expression of several p53 targets, including CDKN1A, SESN1, PUMA, and BAX [6,39].

## 4. Experimental Section

### 4.1. Cell Lines

Primary human fibroblast cell line (F11) was established from skin biopsies taken from foreskin samples of children undergoing circumcision for medical indications, as described previously [10].

### 4.2. Establishment of Stable shTP53inp1 Expressing Cell Lines

According to the manufacturer’s protocol of Santa Cruz Biotechnology shRNA (Santa Cruz Biotechnology, Santa Cruz, CA, USA) with three gene-specific shRNA expression lentiviral vectors (human *TP53inp1* shRNA) transfected into subconfluent F11hT cells. Each vector contains a puromycin resistance gene for the selection of cells stable expressing shRNA. F11hT cells were transfected with *TP53inp1-shRNA* encoded lentivirus (shTP) or control lentivirus (non targeted NT) and cultured at 37 °C. Transduction was performed serum free medium in the presence of 8 μg/mL polybrene (hexadimethrine bromide; Sigma-Aldrich, St. Louis, MO, USA). One hour later the virus-containing media was removed, standard culture media was added, and cultivation is continued at 37 °C. Cells were kept in puromycin selection (2 µg/mL) medium and resistant cells were maintained for 1–2 additional passages. At 70%–80% confluence cells were harvested and lysed. Puromycin resistant cells were selected by long term cultivation to obtain stable transduced F11hT subclones (referred to hereafter as F11hT-shTP).

### 4.3. Radiation Treatment, Colony Forming Assay

Cells were exposed to different doses of ^60^Co γ-rays (Gammatron-3; Siemens, Erlangen, Germany; dose rate; was 0.37 Gy/min) at room temperature. Survival fractions were measured as previously published [8].

### 4.4. RNA Isolation and Real-Time Quantitative PCR (qPCR)

Total RNA was isolated from irradiated and mock-irradiated cells using RNeasy Mini kit (Qiagen, Hilden, Germany) according to the manufacturer’s instructions. To quantify mRNA levels, quantitative real-time PCR was performed using a Rotor-Gene, Corbett-3000 real-time PCR System (Invitrogen, Carlsbad, CA, USA). The standard protocol is previously written [8]. The primer pairs used in PCR studies are shown in Table 1.

**Table 1:** Oligonucleotide primers used in quantitative real time-PCR.

Gene	Forward	Reverse
*TP53INP1*	TCAGCAGAAGAAGAAGAAGAAGAG	AGCAGGAATCACTTGTATCAGC
*CDKN1A*	CCTCATCCCGTGTTCTCCTTT	GTACCACCCAGCGGACAAGT
*GDF-15*	TCACGCCAGAAGTGCGGCTG	CGTCCCACGACCTTGACGCC
*GAPDH*	CGACCACTTTGTCAAGCTCA	AGGGGTCTACATGGCAACTG
*ACTIN*	TTGCCGACAGGATGCAGAAGGA	AGGTGGACAGCGAGGCCAGGAT
*mtDel*	CCCACTGTAAAGCTAACTTAGCATTAACC	GGTTTCGATGATGAGGTCTTTG
*mtWT*	CTGAGCCTTTTACCACTCCAG	GGTGATTGATACTCCTGATGCG

### 4.5. Detection of Autophagic Vacuoles by Acridine Orange

Cells were grown on glass coverslips at 70% confluent cells were treated with 6 Gy. 48 h later, cells were treated with 1 μg/mL acridine orange (were purchased from Sigma-Aldrich Ltd., St. Louis, MO, USA) in serum-free medium for 15 min. The cells were washed with PBS and red fluorescent puncta were observed with AxioImager A1 fluorescence microscope (Carl Zeiss, Oberkochen Jena, Germany). The cytoplasm and nucleus of the stained cells fluoresced bright green according to the hTERT-GFP expression, whereas the AO-positive vacuoles fluoresced bright red. Autophagy was quantified by the mean number of cells displaying intense red dots staining for three fields (measuring at least 50 cells per field) according to Paglin *et al.* [37] for each experimental condition.

### 4.6. Western Blotting

Cell lysis was done with RIPA lysis and extraction buffer (Thermo Scientific, Carlsbad, CA, USA) supplied with Halt protease inhibitor (Thermo Scientific) at 1× final concentration and centrifuged at 14,000× *g* at 4 °C for 15 min. Total protein content was determined by the Bradford protein assay. Samples were loaded on 12.5% Tris-glycine polyacrilamide gels then blotted onto Immuno-Blot PVDF membrane (Bio-Rad Laboratories, Hercules, CA, USA). After blocking, the used primary antibodies were rabbit polyclonal anti-*TP53inp1* (ProteinTech, Manchester, UK) and rabbit polyclonal anti-histone H3 (Santa Cruz, Dallas, TX, USA). Polyclonal donkey anti-rabbit IgG (Abcam, Cambridge, UK) was used as a secondary antibody labeled with HRP. The membrane was treated with Pierce ECL plus substrate (Thermo Scientific) and bands were visualized on standard x-ray film (Kodak, Rochester, NY, USA). Densitometric analysis of the bands, relative to Histone-H3, was performed from the digital images using ImageJ software (http://imagej.nih.gov/ij/, public domain software made by the National Institutes of Health (USA)).

### 4.7. Flow Cytometry ANALYSIS of LC3B

Cells were harvested and kept on ice until processing. Fibroblast were stained with antibodies against autophagosomes, Anti- LC3B (Sigma, St. Louis, MO, USA) and anti-rabbit IgG-Alexa 488 (Biolegend, San Diego, CA, USA). Staining was done at 4 °C for 45 min in 1% FBS/PBS. Labeled cells were analyzed by using FACS Calibur flow cytometer and CellQuest software (BD Biosciences, San Jose, CA, USA). For each sample, an isotype control was used to determine the positive and negative cell populations, and analysis was performed by quadrant analysis.

### 4.8. Senescence-Associated β-Galactosidase Staining

Investigating senescence, *in situ* staining for senescence-associated β-galactosidase (SA-β-gal) was performed [40].

### 4.9. Measurement of Mitochondrial DNA Deletion (CD) by qPCR

DNA isolation from the cells was made with MasterPure DNA Purification kit (EPICENTRE Biotechnologies, Madison, WI, USA). Q-PCR amplifications were carried out with Maxima SYBR Green/ROX Master mix (Fermentas, Vilnius, Lithuania) using mitochondrial DNA-specific primers (Table 1). Reactions were made in duplicate and repeated at least twice from a minimum of three independent biological samples. Expression patterns were normalized relative to (GAPDH) gene, and to the total mitochondrial DNA using the ΔΔ*C*_t_ method in the Rotor-Gene version 6.0.33 software (Corbett Life Sciences, Sidney, Australia).

### 4.10. Data Analysis

The results are shown as the mean and the ±SEM. of at least three independent experiments. Statistical values were calculated using unpaired Student's *t*-test or one-way analysis of variance and two-tailed *t*-tests were used to compare differences among groups. *p* < 0.05 was considered statistically significant (GraphPad Prism 5.0; Software, San Diego, CA, USA). 

## 5. Conclusions

We have presented evidences that γ-radiations induced *TP53inp1* expression in F11hT human fibroblast cells in dose-dependent manner suggesting that *TP53inp1* might serve as a radiation response gene. *TP53inp1* is a p53 target gene and it could regulate the transcription of other p53 targets such as *CDKN1A* and *GDF-15*. Silencing of *TP53inp1* enhanced radiation sensitivity in human fibroblast cells, moreover it increased the frequency of common mitochondrial DNA deletions, while decreased radiation-induced autophagy.
